# Effects of Macronutrient Deprivation on Spring Wheat Productivity

**DOI:** 10.3390/plants15071094

**Published:** 2026-04-02

**Authors:** Ernestas Petrauskas, Lina Skinulienė, Mantas Lukoševičius, Vytautas Petkus, Andrius Stankevičius, Ernestas Zaleckas

**Affiliations:** 1 Department of Agroecosystems and Soil Sciences, Academy of Agriculture, Vytautas Magnus University, K. Donelaičio St. 58, 44248 Kaunas, Lithuania; ernestas@agronom.lt (E.P.); lina.skinuliene@vdu.lt (L.S.); 2 Faculty of Informatics, Kaunas University of Technology, K. Donelaičio St. 73, 44249 Kaunas, Lithuania; mantas.lukosevicius@ktu.lt; 3 Health Telematics Science Institute, Kaunas University of Technology, K. Donelaičio St. 73, 44249 Kaunas, Lithuania; vytautas.petkus@ktu.lt; 4 Department of Aeronautical Engineering, Antanas Gustaitis Aviation Institute, Vilnius Tech University, Linkmenų g. 28, 08217 Vilnius, Lithuania; andrius.stankevicius@vilniustech.lt; 5 Bioeconomy Research Institute, Academy of Agriculture, Vytautas Magnus University, K. Donelaičio St. 58, 44248 Kaunas, Lithuania

**Keywords:** hydroponics, deprivation duration, adaptive response, spectrophotometry, precision agriculture

## Abstract

The aim of this study was to investigate how delayed fertilization with individual macronutrients (N, P, K, Ca, Mg, and S) affects the growth, yield components, biomass, and spectrophotometric indicators of spring wheat grown under controlled hydroponic conditions. Nutrient deprivation was initiated at BBCH stage 23 and maintained for 21, 28, 35, or 133 days, corresponding to BBCH stages 30, 32, 37, and 99, respectively. In selected treatments, the complete nutrient solution was subsequently restored until harvest to evaluate recovery potential. N, P, and Ca deprivation exerted the strongest negative effects on biomass accumulation across all deprivation durations. Compared to the fully supplied control, biomass was reduced by 60% under N deprivation and by 44.5% under P deprivation after 21 days. After 35 days, calcium deprivation resulted in a 97.7% reduction in biomass. Following 133 days of deprivation, biomass was reduced by 98%, 96.8%, and 95.9% under N, calcium, and P deficiencies, respectively. Root mass followed a similar pattern: after 21 days, it decreased by 52.46% (N) and 36.44% (P); after 28 days—by 57.4% (N) and 52.7% (P); after 35 days—by 90.7% (Ca), 66% (N), and 59.1% (P); and after 133 days—by 95.1–90.1% (Ca, N, P). Magnesium deprivation caused substantial reductions in growth parameters, reflecting its central role in chlorophyll structure and photosynthetic efficiency. Sulfur deprivation resulted in moderate but consistent biomass suppression and spectral divergence, indicating its importance in protein synthesis and redox regulation. Short-term deficiencies allowed partial recovery of growth and productivity, whereas long-term deprivation induced pronounced morphological alterations linked to stress adaptation. These effects were further confirmed through in vivo spectral reflectance measurements compared to healthy control plants.

## 1. Introduction

In recent decades, the stability of cereal crop productivity has been increasingly threatened by soil degradation, climate change, and the declining efficiency of fertilizer use [1,2]. Wheat (*Triticum aestivum* L.), one of the most important cereal crops globally [3], is particularly sensitive to fluctuations in mineral nutrient availability throughout the growing season [4,5]. Even moderate deficiencies in key macronutrients such as nitrogen (N) [6], phosphorus (P), potassium (K), sulfur (S), calcium (Ca), or magnesium (Mg) are known to reduce biomass accumulation, impair physiological functions, and ultimately result in yield loss [7].

Plant nutrition and the role of macronutrients for plants are critical, as the macronutrients nitrogen N, P, K, S, Ca, and Mg are essential components of basic biochemical processes. N forms the basis of proteins and chlorophyll, P is part of ATP and nucleic acids, K regulates osmotic pressure and enzyme activity, and Ca and Mg ensure cell wall structure and chlorophyll function [8]. Deficiencies limit photosynthesis and cell growth, leading to lower mass and yield [9,10]. N plays the most critical role among all nutrients, serving as a key component of amino acids, chlorophyll, and enzymes involved in photosynthesis and protein synthesis. N deprivation results in pale green leaves, poor tillering, and reduced plant height and aboveground biomass [10] with a concomitant lack of yield. Since about 75% of leaf N is localized in chloroplasts, its shortage disrupts photosynthetic capacity and promotes early leaf senescence. Early-season N limitation significantly reduces spike number and yield, while late deprivation diminishes grain filling and protein content [11,12,13].

P is vital for energy transfer (ATP), nucleic acid formation, and root development. Deprivation symptoms include dark green or purplish leaves, reduced tillering, and delayed development. Low P availability restricts root elongation, slows cell division, and delays phenological stages, resulting in fewer spikes and reduced biomass. Early-season P deprivation often causes irreversible yield losses, even if later corrected [14,15,16,17].

K regulates osmotic balance, enzyme activation, and carbohydrate translocation. Its deprivation is characterized by marginal chlorosis, tip necrosis, and early senescence of older leaves. Inadequate K supply reduces flag leaf area, shortens the grain-filling period, and decreases both grain number and kernel weight. Moreover, K-deficient plants show lower drought tolerance and reduced photosynthetic performance [17,18,19,20].

S plays a key role in amino acid, protein, and enzyme synthesis. Because S is immobile within the plant, deprivation symptoms appear first on young leaves as uniform chlorosis. S shortage reduces chlorophyll content, delays maturity, and decreases both yield and grain protein concentration. Adequate S enhances N utilization and improves baking quality, which is especially critical in high-yielding cultivars [20,21].

Ca and Mg deficiencies, though less frequent, can cause severe physiological disturbances. Ca is essential for cell wall stability, membrane permeability, and root elongation; its deprivation, common in acidic or sandy soils, leads to root tip necrosis and death of young tissues [22]. Mg, the central atom in the chlorophyll molecule, is vital for photosynthesis and sugar transport. Its deprivation causes interveinal chlorosis on older leaves, reduced CO_2_ assimilation, and impaired carbohydrate translocation, resulting in smaller, lower-quality grains [23,24].

Although the agronomic importance of nutrient supply for wheat cultivation is well recognized, the specific morphological and physiological responses of wheat to deficiencies in individual macronutrients have not yet been sufficiently investigated, especially when stress lasts for different periods of time. Previous studies have focused mainly on yield reduction or general physiological disorders caused by nutrient deprivation, and the deprivation itself has often been assessed as a static condition, without considering the influence of stress duration. However, such studies often fail to take into account the dynamic nature of plant responses, which can include both degenerative effects and adaptive mechanisms designed to ensure survival and reproduction [25,26]. In natural and agricultural environments, nutrient deficiencies often occur periodically, so the effects of short-term and long-term macroelement deficiencies on the morphological and physiological plasticity of wheat remain insufficiently studied. Understanding whether wheat plants simply degenerate in the presence of nutrient deficiencies or whether they can reorganize their metabolic and structural functions to maintain development is very important for both basic plant physiology and applied crop management. Furthermore, there is a lack of systematic comparisons of short-term and long-term nutrient deficiencies covering several essential macronutrients in the literature [27], and the mechanisms that distinguish temporary adaptation responses from irreversible degenerative effects are not yet clearly defined.

The present study aimed to determine how the duration of individual macronutrient deficiencies (N, P, K, S, Ca, Mg) affects the growth dynamics and physiological performance of spring wheat (*Triticum aestivum* L.) under controlled hydroponic conditions. The research evaluated changes in key vegetative parameters, including biomass accumulation, tiller formation, and root development, as well as reproductive indicators such as spike traits, under short- and long-term nutrient deprivation. In addition, the potential for growth recovery after nutrient resupply was assessed to identify the temporal threshold between reversible and irreversible responses.

## 2. Materials and Methods

### 2.1. Plant Material and Growth Conditions

**Figure 1:**
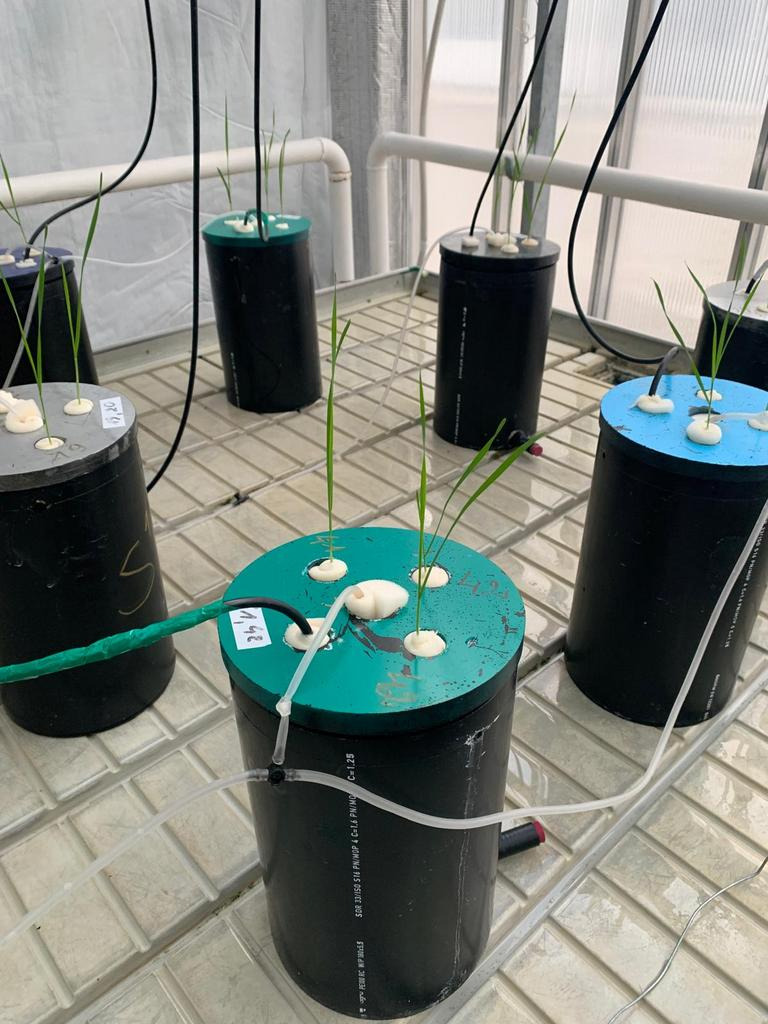
Wheat plants in vegetation tanks.

The experiment was conducted in a controlled-environment greenhouse facility at Vytautas Magnus University, Academy of Agriculture (Akademija, Lithuania, 54°53′ N, 23°50′ E), using a hydroponic growing system. Seeds of spring wheat (*Triticum aestivum* L., cv. Hamlet) were surface-sterilized in 0.2% KMnO_4_ for 30 min and rinsed five times with sterile distilled water. Seeds were placed on moist germination paper for germination under controlled conditions. Germinated seedlings were transferred to foam rubber that was placed in aerated hydroponic tanks fitted with a custom acrylic lid (Figure 1).

A total of 390 uniform spring wheat seedlings were selected and transferred to hydroponic culture, with three seedlings planted per 7.5 L plastic tank (130 tanks in total). The experimental design was completely randomized with three replicates per treatment.

All plants were initially supplied with a Hoagland nutrient solution till BBCH (Biologische Bundesanstalt, Bundessortenamt und Chemische Industrie) stage 23 [28]. Nutrient deprivation began at BBCH 23 (early tillering) and was maintained for 21, 28, 35, or 133 days, corresponding approximately to BBCH stages 30, 32, 37, and 99, respectively. In the 21-, 28-, and 35-day treatments, the omitted macronutrient was subsequently restored, and plants received the complete nutrient solution identical to the control until harvest to assess recovery potential. In contrast, in the 133 days treatment the omitted macronutrient was not resupplied, and plants remained under continuous deprivation from BBCH 23 until final harvest (BBCH 99). Plants that were grown continuously receiving complete modified Hoagland solution served as controls. The structure of deprivation treatments according to duration and recovery regime is presented in Table 1.

Nutrient solutions were renewed every three days to maintain stable chemical conditions and minimize secondary nutrient imbalances. The total experimental duration from BBCH 23 to harvest was 133 days.

The greenhouse conditions were maintained at 22–27 °C during the day and 16–20 °C at night, with a relative humidity of 60–70%. Natural light was supplemented with high-pressure sodium lamps (HPS 400W, Hortilux Schréder, Monster, The Netherlands) to achieve a mean photosynthetically active radiation (PAR) of 400 µmol m^−2^ s^−1^ for 14 h per day, ensuring a uniform photoperiod of 14 h light and 10 h darkness. Oxygenation was maintained by a separate aeration system using air stones and an air pump.

A standard Hoagland solution (modified) was prepared and used as the nutrient medium. The nutrient solution used was a modified Hoagland’s solution composed of 2 mM calcium nitrate (Ca(NO_3_)_2_), 1.5 mM K sulfate (K_2_SO_4_), 0.5 mM magnesium sulfate (MgSO_4_), 0.2 mM monopotassium phosphate (KH_2_PO_4_), and 0.1 mM potassium chloride (KCl) to supply the required macronutrients. Iron was provided in the chelated form as 0.2 mM FeNa-EDTA. Micronutrients were supplied in the following concentrations: 0.1 mM boric acid (H_3_BO_3_), 0.2 mM manganese sulfate (MnSO_4_), 0.5 mM zinc sulfate (ZnSO_4_), 0.2 mM copper sulfate (CuSO_4_), and 0.01 µM ammonium molybdate ((NH_4_)_6_Mo_7_O_24_·4H_2_O). The pH of the solution was adjusted to 5.8 ± 0.1 using HCl or NaOH. All reagents were of analytical grade.

### 2.2. Sampling and Measurements

Morphometric parameters were determined at final harvest (BBCH 99). Plant height at maturity (defined as the distance (cm) from the surface of the container to the beginning of the spike) was determined. The following characteristics were also determined at harvest: number of spikes per plant, number of tillers per plant, spike length, number of spikes per plant and number of spikelets per spike, weight of plants and roots. The four plants from each treatment were selected and separated by their tillers to measure their values manually by using a measuring scale, and an average value was taken. Then, root and shoot dry weight was determined by oven-drying (70 °C, 72 h). Recovery assessment was integrated into the experimental design.

### 2.3. Spectrophotometric Measurements

Spectral reflectance measurements were performed weekly throughout the vegetative period using a Smart Agrometer device (https://www.agronom.lt, accessed on 23 March 2026, JSC “Žemdirbių konsultacijos”, Lithuania). The Smart Agrometer was used to assess plant physiological status by measuring plant reflectance spectral parameters on all developed leaves. Reflectance spectra of each plant were recorded weekly throughout the vegetative period, capturing the spectral response of the plant canopy. Spectral measurements were performed both during the macronutrient deprivation phase and after nutrient resupply, allowing dynamic assessment of physiological divergence and subsequent recovery relative to the control treatment. The calibrated device contains an internal broadband light source and a spectrometer covering visible and near-infrared light (400–850 nm). The device operates using optical sensors, providing real-time data which were recorded to the cloud via mobile network connection and exported for statistical analysis. Calibration and validation were performed according to the manufacturer’s specifications before each use. The commercial Smart Agrometer system uses the same type of measurements (but not algorithms) to predict crop deficiencies. Spectral distances indicate the average differences between crops that can be measured with a reflectance spectrometer.

### 2.4. Statistical Analysis

During the study, the yearly data were processed by applying a one-factor analysis of variance (ANOVA) using the ANOVA module of the SYSTAT software package, version 12. The probability level of differences among the treatments was determined using the LSD test [29,30].

## 3. Results

### 3.1. Yield Components and Productivity

**Figure 2:**
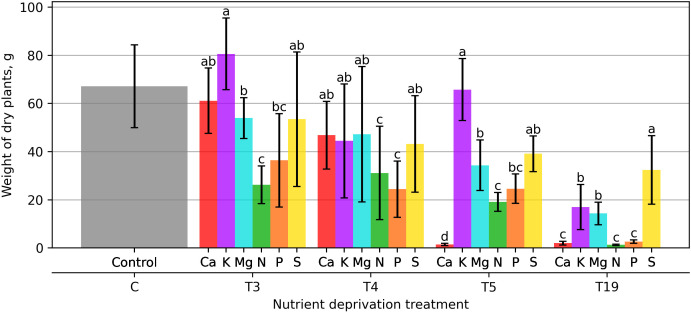
Total weight of dry aboveground biomass. Bars represent mean values ± standard error. Different letters indicate statistically significant differences among treatments within the same deprivation duration according to Fisher’s LSD test (*p* ≤ 0.05).

The lowest biomass was measured in plants after 21 days of N and P deprivation, and it was 60 and 44.5% lower than in the control group, respectively. The smallest difference in biomass was found when applying Ca deprivation, and it was 6.8% lower than in the control group. K deprivation caused the opposite reaction in plants, and in this variant, plants produced 22.9% more biomass than in the control group (Figure 2).

After 28 days of macronutrient deprivation, the greatest decrease in biomass was observed with P deprivation—62.8%, N—52.5%. A very similar variation in biomass was observed with Ca, K, Mg, and S, ranging from 27.9 to 34.0% compared to the control.

After 35 days of macronutrient deprivation, greater differences became apparent, and the greatest reduction in biomass was observed with Ca deprivation, at 97.7%. A large difference in biomass compared to the control was observed with N deprivation—70.9% less biomass was formed compared to the control, and with P deprivation—62.4%. Plants in the variant without K reacted exceptionally, as in this variant, plant biomass increased by 0.2% compared to the control.

After 133 days of macronutrient deprivation, a significant reduction in plant biomass was observed compared with the control. The lowest biomass was found in variants that did not receive N—98% less biomass, Ca—96.8%, P—95.9%. The smallest difference in biomass was observed when plants did not receive S, as biomass production was 50.6% lower compared to the control (Figure 2).

**Figure 3:**
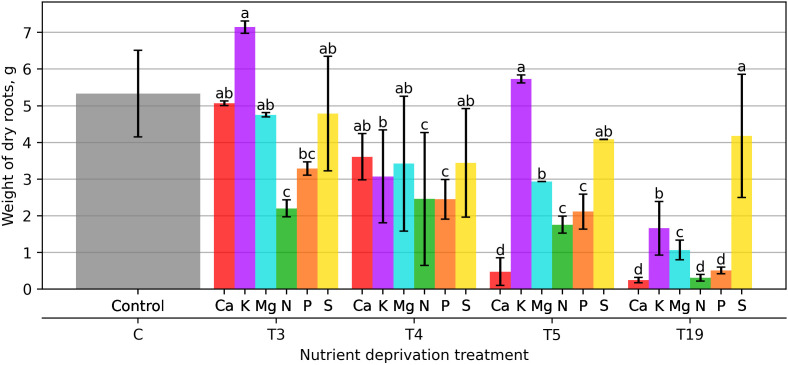
Weight of dry roots under different macronutrient deficiencies and durations. Bars represent mean values ± standard error. Different letters indicate statistically significant differences among treatments within the same deprivation duration according to Fisher’s LSD test (*p* ≤ 0.05).

Plants that experienced a 21 days deprivation of different macronutrients formed different amounts of root mass. Compared with plants receiving a full dose of macronutrients during this period, root biomass decreased by 36.44% and 52.46% under P and N deprivation, respectively, relative to the control (Figure 3). The smallest decrease in root mass was observed in plants with Ca deprivation, which was 2.11%. K deprivation had the opposite effect on plant root mass, which increased by 38%.

Plants that did not receive macronutrients for 28 days from the start of the experiment grew root mass that was relatively uniform among all variants. Ca, S, and Mg deprivation allowed plants to grow 30.3–33.9% less root biomass compared with the control. The greatest reduction in root mass was observed in plants that did not receive N and P, at 57.4% and 52.7%.

After 35 days of macronutrient deprivation, the greatest reduction in root mass was observed in the case of Ca deprivation, at 90.7%, a 66% decrease in N deprivation, and a 59.10% decrease in P deprivation. The smallest decrease in root mass was observed in the case of S deprivation, at 21%. After 35 days of the study, an increase in root mass was observed after 21 days in the case of K deprivation, which was 10.8% compared to the control.

Plants that did not receive macronutrients for 133 days showed the greatest reduction in root mass in the presence of Ca, N, and P, which was between 95.1 and 90.1%. The smallest decrease in root mass compared to the control was observed in the case of S deprivation, 19.2% percent.

Interestingly, plants subjected to 21 days of K or S deprivation were able to compensate later in the growth cycle, achieving root mass values that were comparable to or even greater than those of the control group (Figure 3). This suggests that short-term K or S deprivation may induce overdevelopment of the root system upon recovery.

**Figure 4:**
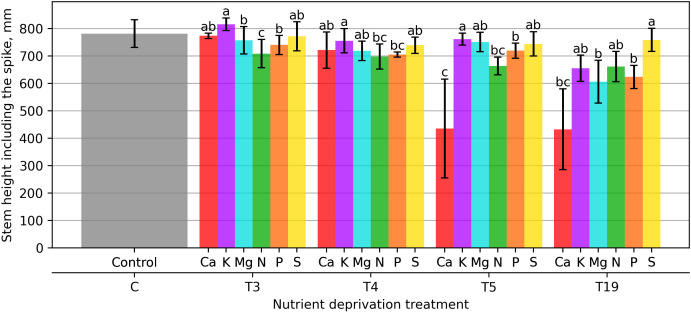
Wheat stem height, including the spike. Bars represent mean values ± standard error. Different letters indicate statistically significant differences among treatments within the same deprivation duration according to Fisher’s LSD test (*p* ≤ 0.05).

The height of reproductive stems was assessed during the study. After 21 days of macronutrient deprivation, it was found that the lowest plants were in the variant that did not receive N and were 10.1% shorter, and those that did not receive P were 6.9% shorter compared to the control (Figure 4). In the variant where the plants experienced a K deprivation for 21 days, the opposite reaction occurred, and the plants became 2.5% taller compared to the control.

A similar trend persisted when macronutrient deprivation was 28 days. The shortest plants were observed under P, N, Mg, and Ca deficiencies (12.9%, 11.3%, 9.4%, and 8.1% shorter than the control, respectively). In contrast, K deprivation had the smallest effect, with plant height only 4.9% lower than the control.

After 35 days, the largest reduction in plant height was observed under Ca deprivation (43.7% vs. control), whereas the smallest difference remained under K deprivation (4.2%).

After 133 days of deprivation, a similar trend remained, i.e., plant height (plants were shorter) differed from the control by only 4% for plants that did not receive S and 13.5% for plants that did not receive N. The most significant difference in plant height was found in plants that did not receive Ca—the plants were 40.5% shorter than the control, Mg—23%, and P—21.2%.

**Figure 5:**
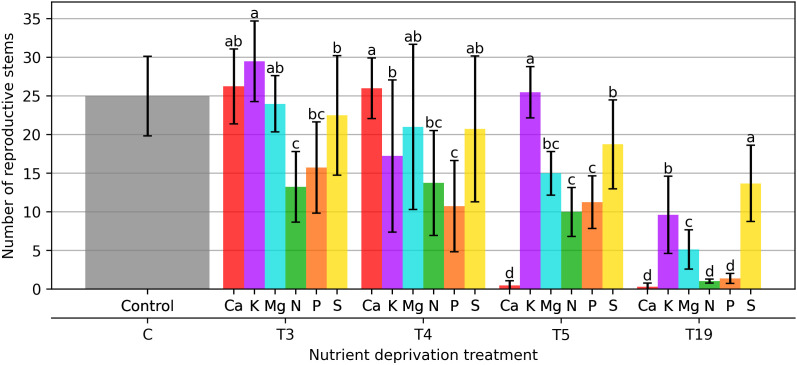
Number of reproductive stems (i.e., those bearing a spike). Bars represent mean values ± standard error. Different letters indicate statistically significant differences among treatments within the same deprivation duration according to Fisher’s LSD test (*p* ≤ 0.05).

The number of reproductive stems was assessed during the study. When the full dose of macronutrients was applied (control), 25 reproductive stems were found (Figure 5). After 21 days of macronutrient deprivation, the plants that reacted the least were those that did not receive Mg, with a 4% decrease compared to the control. Plants that did not receive P and N reacted the most, with the number of reproductive stems decreasing by 37% and 47%, respectively. Variants that experienced Ca and K deprivation reacted differently compared to other variants, with an increase in the number of reproductive stems. In the variant with Ca deprivation, the number of reproductive stems increased by 5%, and in the variant with K deprivation, the number of reproductive stems increased by 18%.

When plants were subjected to a 28 days macronutrient deprivation, the greatest reduction in the number of reproductive stems was observed in the variant with P deprivation, which was a 57% reduction compared to the control. The smallest decrease was observed in the Mg-deficient variant, which was 16% compared to the control. When plants were subjected to a 28 days K deprivation, a significant decrease in reproductive stems was observed, amounting to 31%, while when Ca deprivation was applied, the number of reproductive stems changed by only 1% compared to the 21 days macronutrient deprivation treatment.

When applying a 35 days macronutrient deprivation, the plants reacted very strongly when Ca and K were deficient. A very significant difference was observed when applying Ca deprivation, and the number of reproductive stems decreased by 98% compared to the control. K deprivation caused the opposite reaction, and the number of reproductive stems was found to be 2% higher compared to the control. A stable decline in the number of reproductive stems remained when P and S nutrients were deficient, and it was relatively lower by 55% and 60% compared to the control. After 35 days, Mg deprivation caused a sharp decrease in the number of reproductive stems, which was 40% compared to the control (Figure 5).

The most significant differences were found in plants that did not receive macronutrients for 133 days. The greatest decrease in the number of reproductive stems was observed in plants deprived of Ca (98.8%), N (95.8%), and P (94.4%). Throughout the experiment, S deprivation had the least effect on the number of reproductive stems, with a decrease of 45.2%.

Figure 5 presents the effect of individual macronutrient deficiencies and their duration on the number of reproductive stems (i.e., stems bearing a spike) in spring wheat. Ca deprivation caused the most drastic reduction in reproductive stems. While plants maintained about 25–27 stems up to 28 days of Ca deprivation, the number dropped sharply to approximately 1–2 stems after 35 days and remained at similarly low levels (≈1 stem) after 133 days of deprivation.

A significant decline of reproductive stem number was already evident after 21 days of N deprivation, followed by a continuous decrease with longer deprivation duration. Reproductive stem numbers under K deprivation decreased by about 60% after 133 days compared with the control. This progressive reduction suggests that K plays an important role in sustaining tiller development and reproductive capacity over time.

P deprivation led to a marked reduction in reproductive stems, particularly with increasing duration of deprivation. While short-term P deprivation caused moderate effects, prolonged omission resulted in a sharp decline, with very few reproductive stems remaining after 133 days. P omission reduced the number of stems by 37% after 21 days of deprivation, by approximately 55–57% after 28 and 35 days, and by 94.4% after 133 days.

S deprivation showed a comparatively weaker effect during early stages, with no significant differences from the control up to 21–28 days. However, longer deprivation durations resulted in a significant reduction in the number of reproductive stems, indicating that prolonged S limitation ultimately constrains protein synthesis and tiller fertility, even if early vegetative development appears less affected.

**Figure 6:**
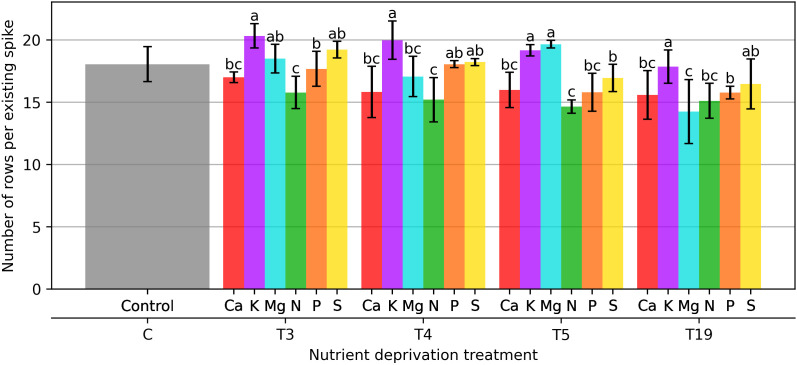
Number of rows per existing spike. Bars represent mean values ± standard error. Different letters indicate statistically significant differences among treatments within the same deprivation duration according to Fisher’s LSD test (*p* ≤ 0.05).

The number of rows per spike (Figure 6), which is a proxy for floret initiation and fertility, remained relatively stable under short-term stress. After evaluating the number of segments after 21 days of macronutrient deprivation, it was found that the variant lacking N had the lowest number of segments, which was 12.2% fewer segments compared to the control. Mg, S and N deficiencies caused an increase in the number of segments from 2.9 to 13.0%.

Plants that lost one of the macronutrients for 28 days had the fewest segments in the variants, lacking N and Ca, 15.4% and 12.0%, respectively. In the variant with K deprivation, the number of segments in the spike increased by 11.1% compared to the control (Figure 6).

The difference in the number of segments was significant after 133 days of macronutrient deprivation. The least response was observed in plants in the variant with K deprivation—0.7%.

The number of rows per spike declined after 133 days of deprivation, particularly in plants deprived of P and S. This decline suggests impaired floral meristem development, potentially caused by energy or redox limitations.

**Figure 7:**
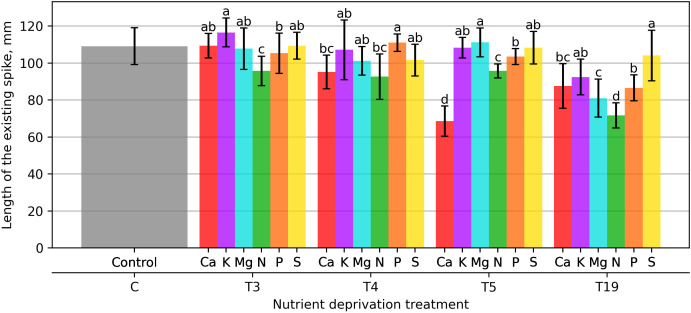
Length of the existing spike (mm). Bars represent mean values ± standard error. Different letters indicate statistically significant differences among treatments within the same deprivation duration according to Fisher’s LSD test (*p* ≤ 0.05).

Plants that experienced a 21 days deprivation of macronutrients formed spikes of varying lengths (Figure 7). Smaller spikes were found in the nitrogen-deficient variant, which were 11.6% smaller than those in the control. Plants with Ca, K, and S deficiencies formed longer spikes by 1%, 7.6%, and 1%, respectively, compared to the control.

Plants with a 28 days macronutrient deprivation formed shorter spikes, but plants without P reacted oppositely and had spikes that were 2.5% longer than the control.

Plants that did not receive macronutrients for 35 days reacted very differently. Ca deprivation prevented plants from forming long spikes, which were 33.7% shorter than the control. Mg deprivation had the opposite effect on plants, with spikes growing 2.7% longer than the control. The least effect was observed in plants with S deprivation, and after 133 days of applied deprivation, the spikes of these plants were 3.9% shorter than the control.

### 3.2. Spectrophotometric Responses and Physiological Signs of Recovery

Spectral distance analysis provided non-invasive and dynamic insights into the physiological state of wheat plants under macronutrient deprivation. By comparing reflectance spectra of treated plants to those of the control over time, we quantified their physiological divergence and recovery potential. Each nutrient deprivation showed a distinct spectral behavior, shaped by both the intensity and duration of the stress.

**Figure 8:**
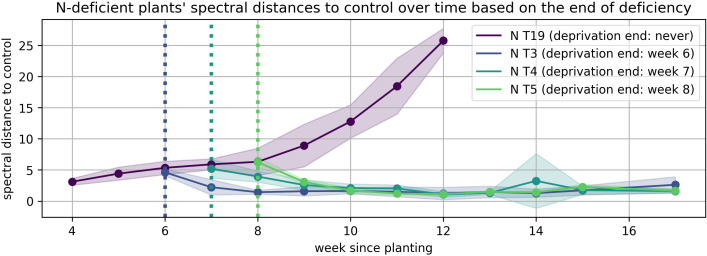
Spectral distance between N-deficient wheat plants and the control group over time.

**Figure 9:**
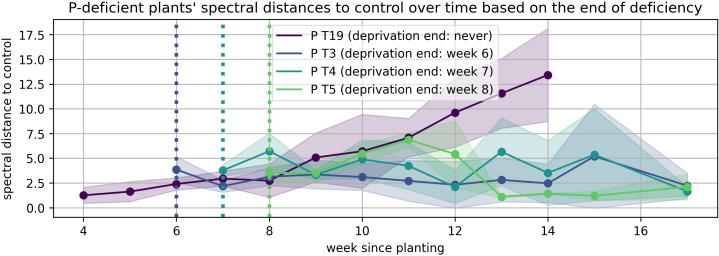
Spectral distance between P-deficient wheat plants and the control group over time.

**Figure 10:**
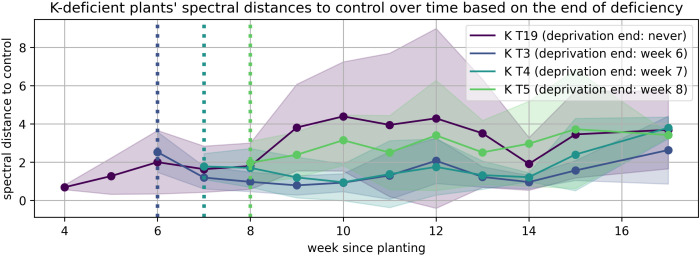
Spectral distance between K-deficient wheat plants and the control group over time.

**Figure 11:**
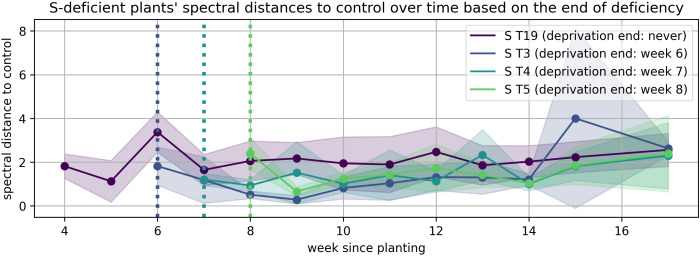
Spectral distance between S-deficient wheat plants and the control group over time.

**Figure 12:**
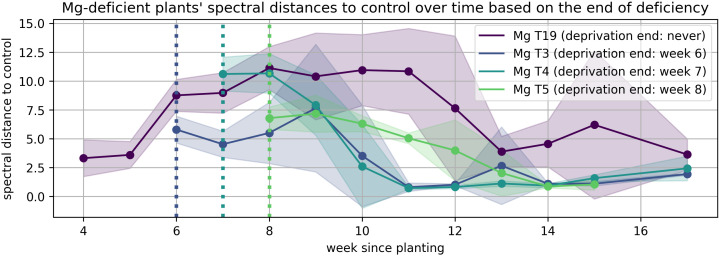
Spectral distance between Mg-deficient wheat plants and the control group over time.

**Figure 13:**
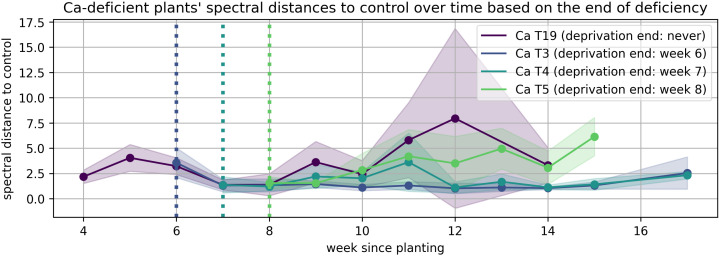
Spectral distance between Ca-deficient wheat plants and the control group over time.

The solid lines in Figure 8, Figure 9, Figure 10, Figure 11, Figure 12, and Figure 13 correspond to the mean distance of the measured reflected spectra of all the developed leaves combined on the treated plants to the healthy control plants of the same week, while the shaded areas of the same color around them indicate the standard deviations of these distances. The dotted vertical lines of the same color indicate the week when the corresponding deficiency ended, as noted in the legend. In Figure 8, Figure 9, Figure 10, Figure 11, Figure 12 and Figure 13 the end of deprivation at vegetation week 6 corresponds to the deprivation duration of 21 days, the end of deprivation at week 7 corresponds to the deprivation duration of 28 days, the end of deprivation at week 8 corresponds to the deprivation duration of 35 days, while the “deprivation end: never” corresponds to the deprivation duration of 133 days (until the harvest) in Figure 2, Figure 3, Figure 4, Figure 5, Figure 6 and Figure 7 and elsewhere in the text.

The results obtained in the case of N deprivation (Figure 8) reveal that plants subjected to prolonged deprivation diverged substantially from the control group, with spectral distances reaching up to 26 distance units by week 12. Not enough plants survived in later days for reliable measurements to be taken. In contrast, plants that experienced N stress for only 21 to 35 days exhibited a steady convergence back to control spectra within 1 to 2 weeks after nutrient re-supply. This indicates a robust physiological recovery, likely mediated by the reactivation of chlorophyll synthesis and restoration of photosynthetic capacity.

Meanwhile, the response to P deprivation (Figure 9) showed a very different trend. Here, spectral distances continued to increase even after P was reintroduced. While plants that were continuously deprived maintained moderate divergence, those that were relieved after three or four weeks still showed delayed and persistent divergence. This suggests that P deprivation may induce irreversible alterations to metabolic or developmental programming.

In the case of K (Figure 10), the observed spectral divergence was moderate. Notably, in all treatments where K was re-supplied, divergence began to decline after week 7. Nevertheless, long-term deprived plants retained higher variability, potentially reflecting the slower recovery of osmotic regulation and cellular ion homeostasis.

A different pattern was observed in cases of S deprivation (Figure 11). The initial spectral divergence was transient and followed by a rapid re-convergence once normal nutrition resumed. This quick recovery shows that S-dependent physiological processes, such as redox regulation and amino acid synthesis, were effectively reactivated upon resupply.

The Mg deprivation treatment (Figure 12) showed a more delayed response. Spectral divergence was highest around weeks 8 to 9 and then gradually reduced. Although the plants showed signs of recovering when Mg was reintroduced after 21 or 28 days, they did not fully recover, which highlights the importance of Mg in maintaining chlorophyll and the fact that the process of restoring their structure is slow.

The results obtained in the case of Ca deprivation (Figure 13) were the least predictable. Even after Ca was added back in, the plants showed unstable and changing patterns of growth. Since Ca plays a very important role in plants—it helps maintain their structural strength and participates in the transmission of signals between cells—its deprivation can have serious consequences. If Ca was lacking for a longer period of time (35 days or more), it affected plant development so severely that it was later impossible to repair the damage. As a result, many plants were unable to grow properly and even died in later stages of growth. Prolonged deprivation of 35 days or more resulted in irreversible damage, with many plants not surviving later growth stages.

The spectrophotometric profiles revealed distinct patterns of physiological reversibility in response to different nutrient deficiencies. Deficiencies in N, S, and K were found to be more readily reversible following short-term deprivation, as plants in these treatments demonstrated rapid spectral re-convergence upon nutrient re-supply. This suggests that the physiological processes affected by N, S, and K—such as chlorophyll synthesis, redox balance, and osmotic regulation—can be effectively restored if the deprivation is promptly corrected.

After restoration of full nutrient supply, newly formed leaves developed a healthy structure, while older leaves exhibited only partial recovery. This pattern reflects the limited reversibility of structural damage in mature tissues. Furthermore, certain deficiencies, such as Ca, appeared to restrict the plant’s capacity to produce new leaves, whereas others, such as S deprivation, allowed continued formation of new tissues after nutrient resupply.

### 3.3. Assessment of Recovery Potential

To quantify the effects of macronutrient deprivation and evaluate the reversibility of stress, key morphometric and productivity parameters were statistically analyzed using LSD post hoc comparisons (α = 0.05). The results allow a nuanced classification of treatments into reversibly affected, partially recovering, or irreversibly suppressed categories (Table 2).

The mean stem height of control plants reached 681.5 mm. Significant reductions (*p* < 0.01) were observed in long-term Ca- and N-deficient treatments, with final heights of 384.0 mm and 405.4 mm, respectively. Short-term deprivation (21 and 35 days) in K and S variants did not differ significantly from the control, indicating potential for full structural recovery.

In green biomass, long-term N and Ca deprivation reduced values by over 50% compared to the control. Mg-deprived plants under 21 days stress maintained biomass within a non-significant distance to the control (*p* > 0.1), supporting tolerance to early Mg deprivation. In contrast, P and S deprivation resulted in reductions in biomass relative to the fully supplied control, especially beyond 28 days.

**Table 2:** Recovery typology based on statistics.

Nutrient	Long-Term Deprivation (133 days)	Short-Term Deprivation (≤35 days)
N	Irreversible suppression	Partial recovery
P	Moderate to severe loss	Minimal recovery
K	Partial suppression	Full recovery
S	Gradual suppression	Nspike-complete recovery
Ca	Irreversible suppression	Weak recovery
Mg	Moderate loss	Full recovery

### 3.4. Morphological and Visual Changes in Wheat Under Delayed Fertilization Conditions

**Figure 14:**
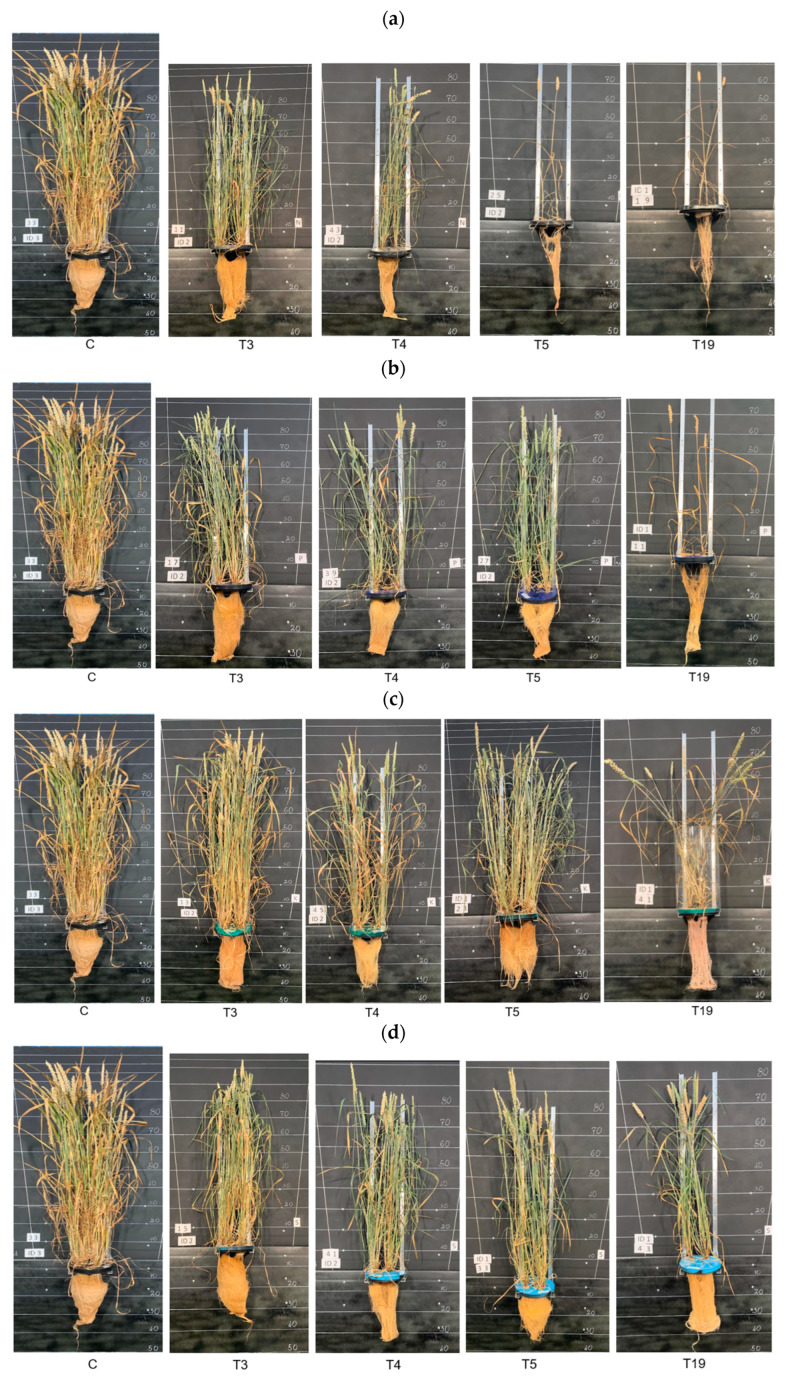

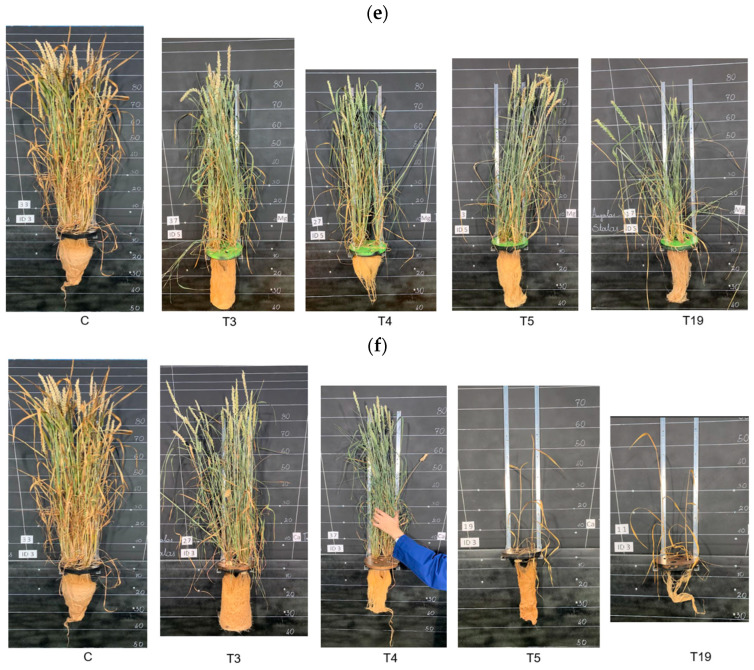
Time-dependent morphological and visual responses of wheat to: (**a**) N, (**b**) P, (**c**) K, (**d**) S, (**e**) Mg and (**f**) Ca deprivation.

Figure 14 illustrates the time-dependent morphological responses of wheat plants subjected to different macronutrient deprivation treatments (N, P, K, S, Mg, and Ca) compared with the fully supplied control. Overall, plant architecture, shoot density, and biomass accumulation progressively declined as the duration of nutrient deprivation increased (21, 28, 35, and 133 days), although the severity of these effects depended on the specific nutrient omitted. N deprivation resulted in the most rapid and pronounced reduction in plant vigor, with progressively decreasing shoot density and simplified canopy structure, particularly after prolonged deprivation (Figure 14a). P deprivation was mainly associated with inhibited plant growth and reduced plant size, with structural decline becoming more evident as deprivation duration increased (Figure 14b). K deprivation caused relatively moderate structural changes during shorter deprivation periods. However, longer deprivation progressively reduced plant height (Figure 14c). S deprivation showed intermediate responses, where short-term deprivation caused only moderate reductions in plant development, whereas extended deprivation led to visible decreases in shoot density and overall plant vigor (Figure 14d).

Mg deprivation resulted in a gradual decline in plant structure over time, particularly affecting older tissues and overall canopy density (Figure 14e). In contrast, Ca deprivation caused pronounced structural damage, including reduced tillering and weakened plant stability, which became increasingly evident under prolonged deprivation (Figure 14f). Overall, the figure demonstrates that the severity of morphological suppression increased with deprivation duration, with nitrogen and Ca deficiencies producing the strongest negative effects on wheat growth and structural development.

## 4. Discussion

The main effects of macronutrient deprivation and subsequent recovery are summarized in Table 1, which provides an overview of the physiological and morphological responses observed in this study. The results demonstrate that both the type of omitted nutrient and the duration of deprivation significantly affected the number of reproductive stems, with clear nutrient-specific response patterns and a strong duration-dependent decline, confirming the interaction between these factors. Root biomass showed more resilient dynamics: in K and S treatments, even 35 days deprivation variants recovered to control-equivalent levels (*p* > 0.5). However, P and Ca stress consistently resulted in significant root mass loss, indicating impaired long-term acquisition potential.

The number of spikelet segments per spike was the most stress-sensitive indicator. Under long-term P or S deprivation, this number dropped from 15.3 (control) to below 7.1 (*p* < 0.001). Conversely, short-term K deprivation had no significant effect (*p* = 0.91), reinforcing the selective plasticity of the reproductive program.

Similarly, spike length was significantly shortened under continuous N or Ca stress, but fully recovered after 21 days Mg deprivation. The number of reproductive stems closely mirrored these trends, with severe suppression under Ca and N, and minimal changes under S and Mg when stress duration was limited.

These statistical results confirm the spectrophotometric findings: N and Ca deprivation have the most pronounced and irreversible effects, while Mg and K stress allow physiological and morphological compensation when addressed early. P and S exhibit intermediate profiles, with partial reversibility depending on timing and developmental stage.

### 4.1. Evidence of Adaptive Responses Under Nutrient Stress

The results of this study confirm the initial hypothesis that the physiological and morphological responses of wheat to macronutrient deprivation were not merely the result of degeneration or growth collapse, but rather represented a structured stress-induced reorganization of plant development. In particular, short-term deficiencies of nutrients such as K, Mg, and S caused temporary suppression of growth, followed by partial recovery in root mass, stem height, and reproductive development. This response pattern indicates that spring wheat exhibits considerable physiological plasticity, allowing plants to redistribute internal resources and maintain reproductive capacity under suboptimal nutritional conditions [31]. Previous studies have also demonstrated that delayed P supply (14–21 days after deprivation) can stimulate shoot growth, improve nutrient uptake, and enhance nutrient use efficiency in wheat. Phosphorus and calcium play essential roles in root meristem activity and cell division; therefore, their absence likely disrupted root elongation and lateral root formation [32]. Similarly, phosphate deprivation in wheat has been reported to increase root length while simultaneously decreasing total P content and reducing both shoot and root biomass [33,34,35]. These findings support the concept that plants actively modify root architecture according to nutrient availability, and that such morphological adjustments can serve as indicators of plant nutritional status.

Previous research has described nutrient deprivation as primarily limiting metabolic functions and biomass accumulation [5,36]. K deprivation can result in reduced growth due to decreased photosynthetic carbon assimilation [37], reduced biomass, and impaired root development [38]. However, our findings highlight the timing-dependent reversibility of such limitations. For example, N deprivation was associated with strong reductions in chlorophyll function and plant height [27], but early re-supply enabled a partial physiological recovery, as detected through spectral convergence. Prolonged deficiencies in N and Ca led to sharp reductions in biomass, likely due to inhibited photosynthesis and reduced vascular transport capacity. Interestingly, Mg-deficient plants maintained relatively high total dry weight under short-term stress, suggesting that initial Mg deprivation does not immediately impair chlorophyll stability or carbon assimilation.

Spectral divergence and convergence patterns observed in this study can be mechanistically linked to well-documented relationships between leaf optical properties and physiological processes. Reflectance in the red and red-edge regions is strongly associated with chlorophyll concentration and N status, whereas near-infrared reflectance is influenced by mesophyll structure and internal light scattering [39,40]. P deprivation may induce metabolic reprogramming and ATP limitation, affecting membrane phospholipid composition and cellular organization, thereby modifying optical scattering properties. In contrast, K and S resupply likely restored osmotic regulation and redox-controlled metabolic pathways, facilitating rapid spectral re-convergence, a phenomenon consistent with hyperspectral stress detection studies in cereals [41,42].

The ability of certain organs or functions to recover, while others remain permanently affected, indicates that wheat plants apply a hierarchical system of priorities under stress conditions. Morphological traits such as total biomass or stem length may be sacrificed to conserve energy for reproductive processes like spike formation and seed row differentiation. This reflects an evolutionary strategy: survival of the genotype via seed production, rather than maximization of biomass.

### 4.2. Duration-Dependent Reversibility and Critical Thresholds

The experimental design allowed clear differentiation between transient and chronic nutrient stress responses. Across all tested macronutrients, the duration of deprivation was the strongest determinant of whether physiological and morphological traits could be restored after resupply.

Plants exposed to 21 days deprivation frequently showed no statistically significant reduction in yield parameters (e.g., biomass, stem number, spike length), and spectrophotometric convergence with the control was rapid. This was especially evident for K, S, and Mg, suggesting that early stress activates adaptive compensation mechanisms without irreversible damage. These mechanisms may include temporary growth suppression, osmotic adjustment, and hormonal modulation [43,44].

However, plants subjected to 133 days continuous deprivation showed consistent and often irreversible suppression of organ development. In Ca and N-deficient plants, not only was recovery absent, but physiological divergence increased over time, indicating compounding damage. This response reflects the critical role of N in tiller initiation, survival, and spike development, as N shortage directly restricts vegetative growth and resource allocation to reproductive organs. Ca structural role in meristematic integrity and N systemic importance for protein synthesis likely explain this vulnerability [5,45].

A critical threshold of approximately 28–35 days of nutrient deprivation was observed, beyond which several yield-related traits (e.g., spikelet rows and productive stems) did not fully recover after nutrient resupply. This period likely coincides with key wheat developmental stages around BBCH 31, when spike differentiation and potential grain number are determined. Nutrient limitation at this stage may therefore impose irreversible constraints on yield formation.

These observations highlight the importance of not only nutrient sufficiency but also timing in fertilization strategies. Even a brief period of deprivation during a critical growth phase may initiate irreversible morphogenetic divergence, particularly in the reproductive organs.

### 4.3. Implications for Nutrient Management and Plant Stress Physiology

The findings of this study have both agronomic and theoretical significance. From a practical standpoint, the results underline the necessity of precise timing in macronutrient delivery during wheat cultivation. In Lithuania, Ca is typically supplied through liming materials (e.g., CaCO_3_ or dolomite) prior to sowing, P is commonly applied before or at sowing as mineral fertilizers, and N is usually split into two or three in-season applications around tillering (BBCH 21–25) and stem elongation (BBCH 30–32) [46,47]. Therefore, complete nutrient deprivation as imposed in this hydroponic experiment does not directly reflect standard fertilization practice. However, temporary nutrient limitation may occur under field conditions due to waterlogging, low soil temperature, restricted root growth, or limited nutrient mobility, which can transiently reduce nutrient availability during critical developmental stages [47,48]. Specifically, the differential reversibility of stress among nutrients suggests the need for nutrient-specific strategies. Although this study was conducted under controlled hydroponic conditions, the observed nutrient-specific response patterns and duration-dependent recovery thresholds provide mechanistic insights that are relevant to soil-based and field systems. Hydroponics enables precise isolation of single-element deficiencies and eliminates confounding soil factors such as adsorption–desorption dynamics (particularly for P), microbial transformations (notably for N), spatial heterogeneity, and diffusion limitations. In field environments, these processes may delay symptom expression or modify recovery kinetics [49]. Nevertheless, element-specific visual signatures (e.g., interveinal chlorosis under Mg deprivation or meristematic deformation under Ca limitation) are expected to remain diagnostically valid. Importantly, the concept of a time-dependent “recovery window” may be even more critical under field conditions, where delayed fertilization during key developmental stages cannot always restore lost morphogenetic potential. For example, N and Ca should be maintained consistently, particularly through early and mid-vegetative stages, due to their low reversibility potential. Arshad et al. (2012) observed that when plants were deficient in Ca, the plants had shorter shoot lengths and lower weights [45].

In contrast, K and S may tolerate short interruptions, provided re-supply is timely. This insight could inform the design of responsive fertilization systems, such as split applications or controlled-release formulations.

From a physiological perspective, the study supports an emerging paradigm in plant stress biology: nutrient deprivation is not merely a passive process of degeneration, but a regulated developmental shift. The ability of wheat to suppress vegetative growth, maintain minimal reproduction, and later partially recover upon re-supply illustrates a stress-adaptive strategy. Such behavior aligns with theories of stress memory, prioritization, and energy allocation [50,51].

Moreover, spectrophotometric analyses revealed that optical signals can precede visible morphological changes, making them valuable for early stress detection [52]. This supports the integration of non-destructive sensing technologies (e.g., hyperspectral reflectance, AI-driven monitoring) into precision agriculture frameworks, especially for managing nutrient dynamics in hydroponic and field-based systems [53]. These findings align with recent advances in hyperspectral crop monitoring demonstrating that nutrient-specific stress signatures can be detected prior to visible morphological symptoms, particularly through red-edge and NIR-sensitive indices [54].

Overall, the study adds to a growing body of evidence that wheat, like many crops, possesses dynamic physiological responses to nutrient limitation. Understanding the recovery potential window and nutrient-specific stress profiles is key to improving both yield stability and resilience under fluctuating environmental and management conditions.

This study demonstrated that wheat (*Triticum aestivum* L.) exhibits diverse physiological and morphological responses to macronutrient deprivation, shaped strongly by both the type of omitted element and the duration of the stress. The key findings are as follows:

Not all nutrient deficiencies had equal effects. N and Ca shortages caused the most severe and largely irreversible suppression of plant development, whereas K, S, and Mg deficiencies allowed partial or full recovery when nutrients were resupplied within 21–35 days. Similarly, Courbet et al. (2021) reported that short-term (10-day) macronutrient deprivation in hydroponically grown wheat had moderate or no negative effect on growth, and S and K deprivation even increased aboveground biomass by about 20%. Their study also indicated substantial remobilization of nutrients within the plant, particularly Mg and Ca, which were strongly translocated from roots to shoots under deficiency conditions [55].

Duration is critical: a stress period longer than 28–35 days often exceeded the plant’s recovery capacity. Short-term deprivation triggered adaptive growth suppression, whereas prolonged stress led to developmental reprogramming that could not be reversed.

Plants actively adapt: the results support the hypothesis that physiological changes under nutrient stress are not simply degenerative, but represent regulated responses to enhance survival and reproductive assurance. Traits such as root development, spike architecture, and biomass allocation were modulated according to stress severity and timing.

Spectral reflectance is a sensitive indicator: spectrophotometric divergence and convergence patterns reflected physiological stress and recovery dynamics spikier than visible traits, confirming their potential for early stress detection.

Agronomic relevance: findings underline the need for timing-optimized, nutrient-specific fertilization strategies, particularly under hydroponic and controlled-environment conditions, where nutrient dynamics are tightly linked to developmental stages.

### 4.4. Impact of Delayed Fertilization on Wheat Morphological and Visual Traits

The results of this study demonstrate that both the type of macronutrient deprivation and the duration of stress strongly determined the reversibility of growth suppression and the final morphological outcome of wheat plants. Prolonged deficiencies of N and Ca caused the most severe and persistent reductions in biomass accumulation, canopy structure, and reproductive stem formation, reflecting the fundamental roles of N in photosynthetic metabolism and protein synthesis and of Ca in meristem stability and cell wall integrity. In contrast, deficiencies of K, S, and Mg showed greater recovery potential when nutrient supply was restored at earlier stages, indicating that wheat plants can partially compensate for temporary limitations of these elements. However, when deprivation persisted beyond approximately 28 and 35 days, several yield-related traits, including spike architecture and reproductive stem formation, did not fully recover even after nutrient resupply. These findings highlight that not only the identity of the limiting nutrient but also the duration of deprivation determines whether wheat plants can maintain developmental plasticity or undergo irreversible morphological changes.

## 5. Conclusions

This study demonstrates that wheat responses to macronutrient deprivation are strongly dependent on both the specific element omitted and the duration of deprivation. N, P, and Ca deprivation exerted the most severe negative effects on growth and biomass accumulation, particularly under prolonged exposure, cessation of shoot and biomass accumulation. In contrast, K deprivation exhibited a dual response: short-term deprivation stimulated certain biometric traits relative to the control, whereas prolonged deprivation resulted in moderate biomass reduction compared with other macronutrients. S and Mg deprivation caused comparatively milder reductions and allowed partial compensation following nutrient resupply.

The reversibility of nutrient stress was closely linked to both deprivation duration and the functional role of the nutrient in structural versus metabolic processes. Short-term deprivation (21–35 days) of K, Mg, and S permitted substantial recovery after resupply, whereas prolonged deprivation of N and Ca led to largely irreversible impairment of vegetative development.

A deprivation duration of approximately 28–25 days appears to represent a physiological threshold beyond which compensatory growth mechanisms decline and irreversible morphogenetic reprogramming is initiated. Importantly, spectrophotometric changes preceded visible morphological symptoms, highlighting the sensitivity of optical sensing approaches for early detection and real-time monitoring of nutrient stress and recovery dynamics. Overall, the findings emphasize that early macronutrient deprivation can impose long-term developmental constraints even after full resupply, underscoring the importance of timely nutrient management in controlled hydroponic systems.

## Figures and Tables

**Table 1:** Structure of macronutrient deprivation treatments and recovery regimes.

Treatment Code	Duration of Macronutrient Deprivation (Days from BBCH 23)	Nutrient Resupply	Description
C	0 (control)	Continuous full nutrition	Plants received complete nutrient solution throughout the experiment
T3	21	Resupply applied	21 days deprivation followed by full nutrient resupply
T4	28	Resupply applied	28 days deprivation followed by full nutrient resupply
T5	35	Resupply applied	35 days deprivation followed by full nutrient resupply
T19	133	No resupply	Continuous deprivation until harvest (BBCH 99)

## Data Availability

The original contributions presented in this study are included in the article. Further inquiries can be directed to the corresponding author.
